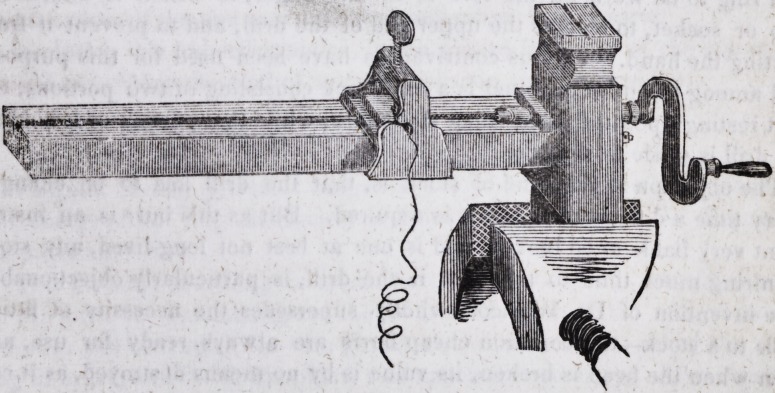# Spiral Springs for Artificial Teeth

**Published:** 1846-12

**Authors:** 


*Spiral Springs for Artificial Teeth.*—The above cut represents an instrument invented by N. Howcott, dentist, of Memphis, Tenn., for winding spiral springs. It is the most perfect contrivance for the purpose which we have ever seen. It is simple in its construction, and its great superiority over every other means which have been employed for coiling wire, consists in the accuracy with which a spring may be wound in it.

As the reader will be able to form a correct idea of its construction and mode of action, from the cut, it is not necessary to describe it. It may be obtained from Mr. F. Arnold, Dental Instrument Manufacturer of Baltimore. *Balt. Ed.*

## Figures and Tables

**Figure f1:**